# Real-World Effectiveness of Capecitabine and Temozolomide Across Endocrine and Neuroendocrine Neoplasm Subtypes (ENENs): A Population-Based Cohort Study from Alberta, Canada (2011–2021)

**DOI:** 10.3390/curroncol33050289

**Published:** 2026-05-14

**Authors:** Alda Aleksi, Kaiden D. Jobin, Malek B. Hannouf, Patrik Husi, Heather Halperin, Chris White-Gloria, Tasnima Abedin, Dean Ruether

**Affiliations:** 1 Cumming School of Medicine, University of Calgary, Calgary, AB T2N 4N1, Canada; 2 Department of Medical Oncology, Arthur J.E. Child Comprehensive Cancer Centre, Calgary, AB T2N 5G2, Canada; 3 Department of Cellular, Molecular and Microbial Biology, University of Calgary, Calgary, AB T2N 4N1, Canada; 4 Department of Medicine, University of Calgary, Calgary, AB T2N 4N1, Canada; 5 Department of Biochemistry, University of Calgary, Calgary, AB T2N 4N1, Canada; 6 Department of Statistics, University of Calgary, Calgary, AB T2N 4N1, Canada

**Keywords:** neuroendocrine neoplasms, endocrine neoplasms, CAPTEM, real-world, retrospective cohort, overall survival, progression-free survival

## Abstract

Capecitabine plus temozolomide (CAPTEM) improves progression-free survival (PFS) in pancreatic endocrine and neuroendocrine neoplasms (PNENs), yet its real-world effectiveness across other endocrine and neuroendocrine neoplasm (ENEN) subtypes remains uncertain. We conducted a retrospective population-based cohort study including 159 adults with ENENs treated with CAPTEM in Alberta, Canada (2011–2021). Patients were stratified by primary tumor site, treatment line, and number of CAPTEM cycles received. Kaplan–Meier methods and Cox proportional hazards models adjusted for age and sex were used to evaluate PFS and overall survival (OS). Compared with pancreatic neuroendocrine neoplasms PNENs, gastrointestinal neuroendocrine neoplasms (GINENs) demonstrated similar PFS and OS. In contrast, pulmonary neuroendocrine neoplasms (PuNENs) and other ENENs (OENENs) were associated with significantly shorter PFS and OS. Use of CAPTEM in the first-line setting was associated with improved PFS (HR 0.56, 95% CI 0.40–0.78, *p* < 0.001) and OS (HR 0.42, 95% CI 0.29–0.62, *p* < 0.001). Receipt of ≥6 treatment cycles was also strongly associated with superior PFS (HR 0.22, 95% CI 0.16–0.32, *p* < 0.001) and OS (HR 0.22, 95% CI 0.14–0.34, *p* < 0.001). CAPTEM shows comparable real-world effectiveness in GINENs and PNENs but appears less effective in PuNENs and other OENEN subtypes. Early initiation and adequate treatment duration are key factors associated with improved survival outcomes.

## Simple Summary

The oral chemotherapy combination of capecitabine and temozolomide (CAPTEM) is widely used for metastatic pancreatic neuroendocrine neoplasms (PNENs), but evidence supporting its use in other endocrine and neuroendocrine neoplasms (ENEN) subtypes remains limited. We conducted a population-based real-world study evaluating survival outcomes among patients with ENENs treated with CAPTEM in Alberta, Canada. Over a 10-year period, CAPTEM demonstrated comparable effectiveness in gastrointestinal neuroendocrine neoplasms relative to PNENs, but inferior outcomes in pulmonary neuroendocrine neoplasms and other non-gastroenteropancreatic endocrine & neuroendocrine neoplasms, ENENs. Importantly, administration of CAPTEM in the first-line setting and delivery of at least six treatment cycles were strongly associated with improved progression-free and overall survival. These findings support broader but more selective use of CAPTEM across ENEN subtypes and highlight treatment timing and duration as clinically relevant prognostic factors in routine oncology practice.

## 1. Background

Endocrine and neuroendocrine neoplasms (ENENs) are defined as tumors demonstrating differentiation towards endocrine or neuroendocrine secretory cell lineages, reflecting their origin from cells capable of hormone production and regulated secretory activity. In accordance with principles reflected in the World Health Organization Classification of Tumors and International Agency for Research on Cancer frameworks, these neoplasms can be grouped based on evidence of endocrine or neuroendocrine differentiation rather than strict adherence to organ-specific taxonomies [1]. This classification approach acknowledges the shared biological and molecular characteristics across tumors arising in different anatomical locations but exhibiting similar differentiation patterns. The most common primary sites include the gastrointestinal tract, pancreas, and bronchopulmonary system, although ENENs may arise in virtually any organ system [2]. Over recent decades, the global incidence and prevalence of ENENs have increased substantially, a trend attributed to improved diagnostic modalities such as advanced imaging and endoscopic techniques, heightened clinical awareness among healthcare providers, evolving pathological classification systems with more precise criteria, and potentially changing environmental or genetic risk factors that remain incompletely understood [3,4,5,6].

ENENs encompass a broad spectrum of clinical behavior, ranging from indolent, well-differentiated tumors associated with prolonged survival and relatively stable disease courses to highly aggressive neoplasms characterized by rapid progression, poor differentiation, and early metastatic dissemination [7]. This marked heterogeneity in tumor biology and clinical trajectory presents significant therapeutic challenges in routine practice, necessitating individualized and multidisciplinary treatment strategies. Management decisions are typically guided by multiple factors, including tumor grade, degree of differentiation, disease burden and distribution, functional status (hormone secretion), somatostatin receptor expression, and patient-related considerations such as comorbidities and performance status.

Systemic treatment options for advanced ENENs have expanded considerably and now include somatostatin analogues for symptom control and tumor stabilization, targeted therapies such as everolimus, sunitinib, and cabozantinib, peptide receptor radionuclide therapy (PRRT), and cytotoxic chemotherapy [8,9]. While cytotoxic regimens have traditionally been reserved for higher-grade tumors or for progressive disease refractory to other systemic therapies, increasing evidence supports their role in selected patients with well-differentiated ENENs who exhibit high tumor burden, aggressive clinical behavior, or rapid disease progression despite other treatments [10,11,12]. These evolving treatment paradigms reflect a growing recognition of the need for flexible and patient-specific approaches.

The oral combination of capecitabine and temozolomide (CAPTEM) has emerged as one of the most widely utilized chemotherapy regimens in metastatic pancreatic neuroendocrine neoplasms (PNENs), owing to its favorable efficacy and tolerability profile. The randomized ECOG-ACRIN E2211 trial demonstrated significantly improved progression-free survival (PFS) with CAPTEM compared with temozolomide monotherapy, thereby establishing CAPTEM as a standard treatment option in this disease setting [10]. However, evidence supporting its use in non-pancreatic ENEN subtypes, including gastrointestinal neuroendocrine neoplasms (GINENs) and bronchopulmonary neuroendocrine neoplasms (PuNENs), remains limited and is largely derived from small retrospective studies with heterogeneous patient populations, variable tumor characteristics, and inconsistent treatment sequencing strategies [13,14,15].

Real-world data are therefore critical to understanding treatment effectiveness outside the controlled environment of clinical trials, particularly in rare malignancies such as ENENs, where large randomized studies are difficult to conduct, and evidence gaps persist. Population-based analyses can provide valuable insights into real-world treatment patterns, prognostic factors, healthcare utilization, and survival outcomes across diverse clinical settings and patient populations.

Accordingly, the objective of this study was to evaluate real-world survival outcomes associated with CAPTEM across multiple ENEN subtypes using comprehensive provincial cancer registry data from Alberta, Canada, spanning the period from 2011 to 2021. In addition, we aimed to assess the impact of treatment timing, sequencing, and duration on clinical outcomes in order to better inform optimal therapeutic strategies in routine clinical practice.

## 2. Materials and Methods

### 2.1. Study Design and Setting

We conducted a retrospective, population-based cohort study evaluating adult patients diagnosed with ENENs who received treatment with the CAPTEM regimen in Alberta between 2011 and 2021. The province operates a publicly funded, single-payer, and highly centralized cancer care system, in which oncology services are delivered through coordinated provincial programs. This structure enables comprehensive capture of diagnostic, treatment, and outcomes data across the entire population, thereby minimizing referral bias and loss to follow-up. Data sources include linked administrative health databases, pharmacy records, and provincial cancer registry datasets, which collectively provide detailed longitudinal information on patient demographics, tumor characteristics, systemic therapies, and survival outcomes.

For the purposes of this study, we defined a composite group of ENENs based on shared differentiation along hormone-producing cellular lineages rather than strictly by anatomical site or traditional histologic classification. Specifically, tumors were included if they arise from, or demonstrate differentiation toward, endocrine or neuroendocrine secretory cells characterized by regulated secretory capacity and expression of lineage-associated molecular programs. This definition encompasses a broad spectrum of neoplasms, including well-differentiated neuroendocrine tumors (NETs; grades 1–3), bronchial carcinoids (typical and atypical), poorly differentiated neuroendocrine carcinomas (including small cell and large cell subtypes), Merkel cell carcinoma, neoplasms of unknown primary and selected endocrine tumors such as thyroid carcinomas, pheochromocytomas, and paragangliomas. This biologically informed grouping reflects shared cellular origin and functional characteristics, while acknowledging substantial heterogeneity in morphology, molecular profiles, clinical behavior, and prognosis across included tumor types.

### 2.2. Data Sources

Patients, tumor, and treatment characteristics were retrospectively retrieved through detailed review of electronic medical records from the Alberta Health Services (AHS) databases, Alberta, Canada, including the Alberta Cancer Registry (ACR), the Cancer Centre Electronic Medical Record (ARIA-MO), the Discharge Abstract Database (DAD), and the National Ambulatory Care Reporting System (NACRS). These linked administrative and clinical data sources provide a comprehensive and population-level repository of oncology-related information across the province. The ACR and ARIA-MO together form an integrated data platform capturing all cancer patients diagnosed and treated in Alberta, and include detailed information on patient demographics, tumor characteristics, surgical interventions, Cancer Control Alberta clinic encounters, radiation therapy delivery, systemic treatments administered, and longitudinal vital status outcomes.

Variables extracted from the ACR included age at diagnosis, sex, and key tumor characteristics such as stage, defined according to the American Joint Committee on Cancer (AJCC) 7th edition staging system, histological subtype, and Ki67 index. Additional variables included vital status, date and cause of death, and date of last known contact with AHS or Cancer Control Alberta, Canada for patients who were not deceased at the time of data cutoff. To supplement registry-derived data, a comprehensive chart review of ARIA-MO records was conducted to obtain granular treatment-related information, including the specific type of chemotherapy regimen, number of cycles administered, and line of therapy. Information on disease recurrence or diagnosis of a new primary malignancy, including date and tumor type, was also captured.

Therapy line categorization was defined based on prior systemic treatment exposure. First-line CAPTEM was defined as administration in patients with no prior systemic therapies. Second-line CAPTEM was defined as treatment following at least one prior systemic regimen or as a rechallenge after a minimum interval of six months since prior CAPTEM exposure, reflecting clinically meaningful treatment sequencing.

### 2.3. Study Population

We included patients who were greater than 18 years of age, diagnosed with an ENEN, and treated with at least one cycle of CAPTEM during the period from January 2011 to December 2021 in Alberta. Eligibility was restricted to individuals with advanced, unresectable, or metastatic disease at the time of CAPTEM initiation, ensuring a clinically homogeneous cohort reflective of real-world treatment indications for systemic chemotherapy. Patients were identified through comprehensive provincial cancer databases, allowing for inclusion of cases managed across both academic and community oncology settings.

We stratified patients into clinically relevant subgroups based on primary tumor site, including PNENs, GINENs, PuNENs, and other ENENs (OENENs), which encompassed less common or atypical primary sites. Additional stratification variables included line of therapy (i.e., CAPTEM administered as first-line versus later-line treatment) and total number of CAPTEM cycles received (i.e., fewer than 6 cycles versus 6 or more cycles). The decision to administer a standard six-cycle course versus extended treatment (up to 12 cycles) was made at the discretion of the treating medical oncologist, reflecting individualized clinical judgment. Continuation beyond six cycles was typically guided by serial radiologic assessment, with treatment extension permitted in cases demonstrating objective response or radiographic disease stability on follow-up imaging, provided there were no significant or treatment-limiting toxicities.

Initiation of CAPTEM chemotherapy was primarily driven by evidence of symptomatic and/or radiologic disease progression, particularly among patients with moderate to high metastatic burden and those with visceral involvement, including cases at risk of impending visceral crisis. In this context, Ki-67 proliferation index had a comparatively limited influence on treatment selection, especially in later lines of therapy where clinical behavior often superseded histologic grading. Notably, the study cohort demonstrated a relatively balanced distribution of tumors across the three Ki-67–defined subgroups, consistent with World Health Organization classification thresholds.

During the study period (2011–2021), dihydropyrimidine dehydrogenase (DPYD) testing was neither mandatory nor publicly funded in Alberta; consequently, routine pre-treatment DPYD screening prior to capecitabine initiation was not performed. DPYD testing only became mandatory and provincially funded in late 2025. Similarly, O6-methylguanine-DNA methyltransferase (MGMT) testing was not available or publicly funded for ENENs in Alberta during the study period and continues to lack public funding for ENENs, limiting its role in guiding treatment decisions within this cohort.

### 2.4. Ethics

The study was approved by the Health Research Ethics Board of Alberta—Cancer Committee (HREBA.CC-22-0219) on 14 June 2023, following institutional review of study design, data sources, and patient privacy considerations. The study was conducted in accordance with STROBE reporting guidelines [16], ensuring transparency, methodological rigor, and completeness in observational research reporting.

### 2.5. Outcome Measures

The primary endpoints of this study were 6-year overall survival (OS) and 6-year progression-free survival (PFS), selected to reflect both long-term survival outcomes and disease control in patients treated with CAPTEM. Disease progression was systematically monitored through serial cross-sectional imaging using computed tomography (CT) or magnetic resonance imaging (MRI), typically performed at intervals of every 3–6 months in accordance with standard clinical practice. PFS was calculated from the date of CAPTEM initiation to the earliest occurrence of radiological disease progression, death from any cause, or last known date alive for censored patients. Overall survival (OS) was defined as the time from CAPTEM initiation to death from any cause or last known follow-up. Radiological progression was defined according to RECIST criteria as a ≥20% increase in the sum of target lesion diameters, or alternatively based on documented clinical assessment in cases where confirmatory imaging was not available [17].

### 2.6. Statistical Analysis

Descriptive statistics were used to summarize baseline patient demographics and disease characteristics across the study cohort. Continuous variables were reported as means with standard deviations or as medians with interquartile ranges, as appropriate based on the underlying data distribution and assessment of normality. Categorical variables were summarized as frequencies and proportions, providing an overview of cohort composition. Comparisons between subgroups were performed using the chi-square test or Fisher’s exact test where applicable, depending on expected cell counts and distributional assumptions.

Time-to-event outcomes, including progression-free survival (PFS) and overall survival (OS), were estimated using the Kaplan–Meier method, allowing for appropriate handling of censored observations. Median survival times and corresponding survival probabilities at prespecified time points were calculated. Differences between survival curves across subgroups were assessed using the log-rank test. Cox proportional hazards regression models were used to estimate hazard ratios (HRs) with corresponding 95% confidence intervals (CIs), providing measures of relative risk over time.

Multivariable Cox proportional hazards models were constructed to evaluate associations between relevant covariates and survival outcomes while adjusting for prespecified confounders, including age and sex. Additional clinically relevant variables were considered where appropriate. The proportional hazards assumption was assessed through graphical evaluation of Schoenfeld residuals to ensure model validity.

All analyses were conducted using SAS software, version 9.4M9 (Cary, NC, USA). All statistical tests were two-sided, and a predefined significance threshold of *p* < 0.05 was applied to determine statistical significance.

## 3. Results

A total of 159 patients diagnosed with ENENs in Alberta between 2011 and 2021 who received at least one cycle of CAPTEM and had complete clinical and treatment data available were included in the final analysis. This cohort represents a real-world population treated within a comprehensive provincial cancer care system. Among these patients, 101 (64%) received CAPTEM in the first-line setting, while 58 (36%) were treated in later lines of therapy following prior systemic treatment exposure (Table 1), reflecting variation in treatment sequencing.

With respect to primary tumor site, 67 patients (42%) had PNENs, 38 (25%) had GINENs, 35 (20%) had PuNENs, and 19 (13%) had OENENs (Table 2). These distributions reflect the relative frequency of tumor origins within the study cohort. Age, sex, line of CAPTEM therapy at initiation, and other key demographic and clinicopathologic characteristics for each subgroup are summarized in Table 1 and Table 2, providing a comprehensive overview of baseline patient and disease features across tumor types.

### 3.1. Survival Outcomes by Tumor Site

**Figure 1:**
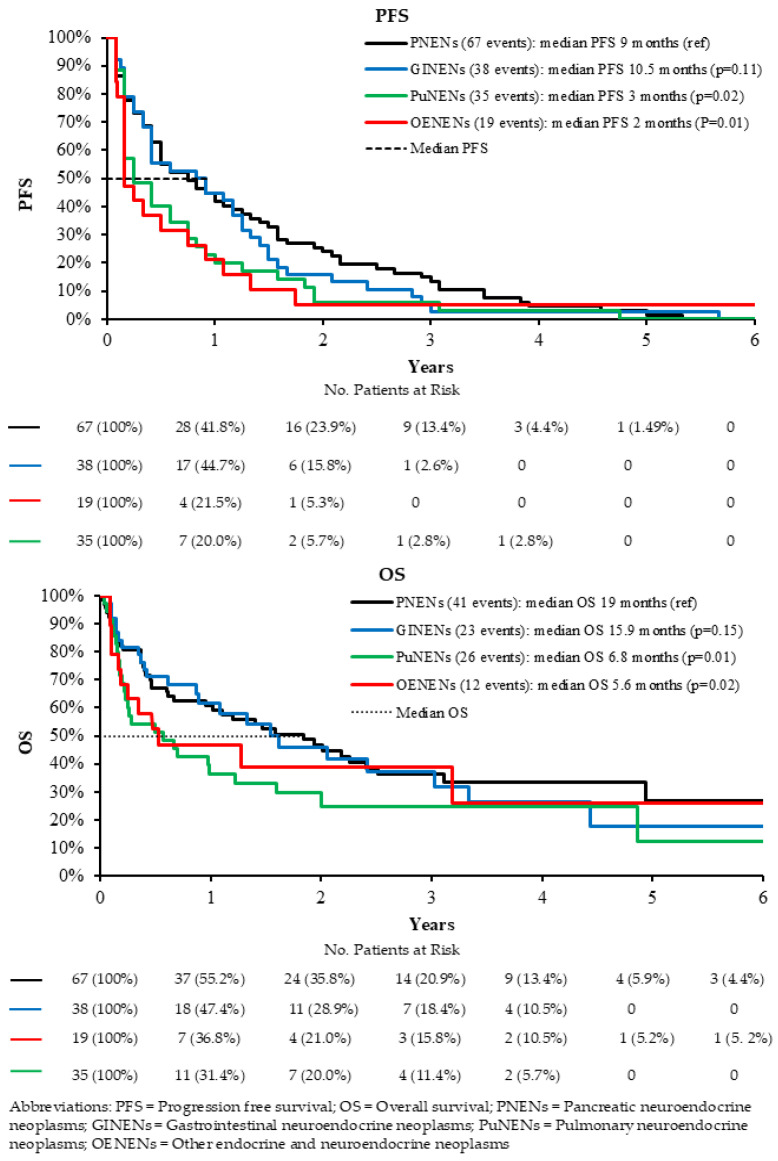
Survival analyses comparing PuNENs, GINENs, and OENENs with PNENs.

When stratified by tumor origin, patients with GINENs demonstrated survival outcomes broadly comparable to those observed in patients with PNENs. In terms of progression-free survival (PFS), the median PFS was 10.5 months in the GINEN cohort compared with 9.0 months in the PNEN cohort (adjusted HR 1.10, 95% CI 0.79–1.65, *p* = 0.101; unadjusted available in Table 3). These findings suggest no statistically significant difference in disease progression risk between these two groups, although a modest numerical difference was observed. Similarly, overall survival (OS) outcomes followed a comparable pattern. Median OS was 15.9 months in patients with GINENs versus 19.0 months in those with PNENs (adjusted HR 1.11, 95% CI 0.69–1.81, *p* = 0.135; unadjusted available in Table 3) (Figure 1, Table 3). Although PNENs showed a numerically longer median OS, the difference did not reach statistical significance, reinforcing the overall similarity in outcomes between these tumor types.

In contrast, patients with PuNENs experienced significantly inferior clinical outcomes when compared to those with PNENs. The disparity was particularly evident in progression-free survival, where median PFS was markedly shorter at 3.0 months in the PuNEN group versus 9.0 months in the PNEN group (adjusted HR 1.65, 95% CI 1.15–2.51, *p* = 0.021; unadjusted available in Table 3). This indicates a substantially higher risk of disease progression among patients with PuNENs. Consistent with this finding, overall survival was also significantly reduced, with a median OS of 6.8 months in PuNENs compared with 19.0 months in PNENs (adjusted HR 1.73, 95% CI 1.08–2.71, *p* = 0.014; unadjusted available in Table 3) (Figure 1, Table 3). These results highlight the more aggressive clinical course associated with PuNENs.

Similarly, patients with OENENs demonstrated significantly poorer survival outcomes relative to PNENs across both key endpoints. Median PFS was notably shorter at 2.0 months compared to 9.0 months in the PNEN cohort (adjusted HR 1.59, 95% CI 1.14–2.74, *p* = 0.020; unadjusted available in Table 3). In addition, median OS was reduced to 5.6 months in patients with OENENs, compared with 19.0 months in PNENs (adjusted HR 1.51, 95% CI 1.05–2.80, *p* = 0.026; unadjusted available in Table 3) (Figure 1, Table 3). Together, these findings emphasize the markedly worse prognosis associated with both PuNENs and OENENs when compared with PNENs, in contrast to the relatively similar outcomes observed between GINENs and PNENs.

### 3.2. Impact of Treatment Line

Across the full cohort, administration of CAPTEM in the first-line setting was associated with significantly improved survival outcomes compared with its use in later-line settings. Patients who received CAPTEM as an initial systemic therapy experienced more favorable disease control and longer survival durations overall. Specifically, median progression-free survival (PFS) was 10.0 months for first-line treatment versus 3.5 months for later-line treatment (HR 0.56, 95% CI 0.40–0.78, *p* < 0.001), indicating a substantially reduced risk of disease progression when CAPTEM was used earlier in the treatment course.

**Figure 2:**
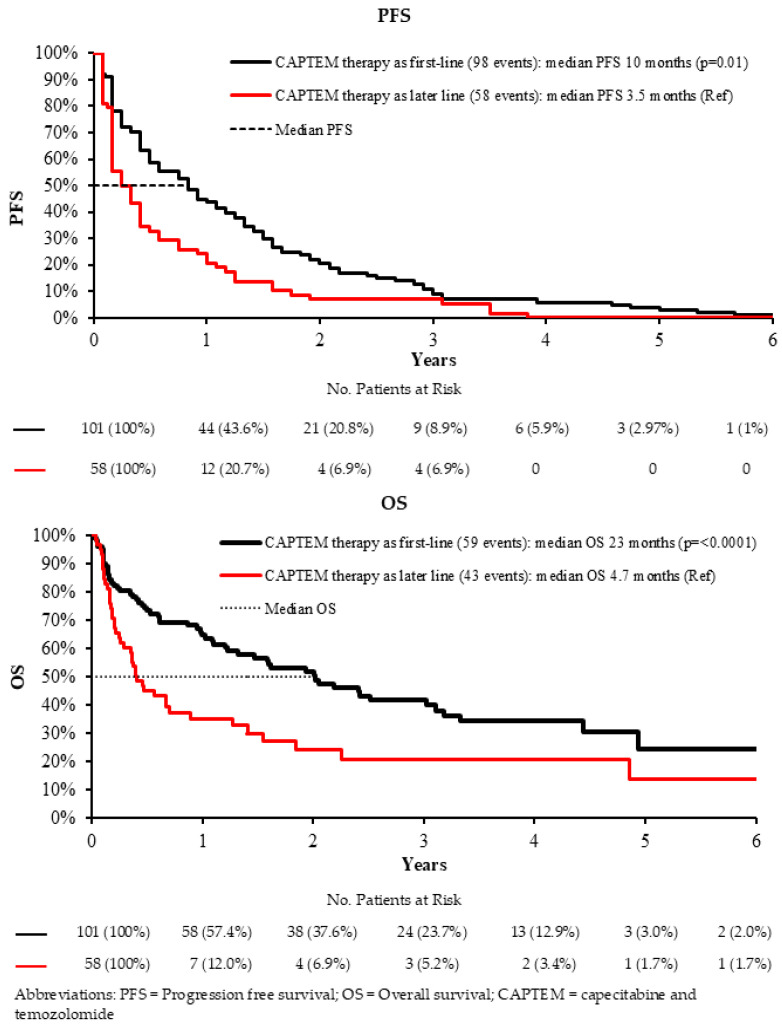
Survival analyses comparing CAPTEM therapy as first-line treatment compared to later-line use.

Median overall survival (OS) was also notably prolonged in the first-line group, reaching 23.7 months compared with only 4.7 months in patients treated in later lines (HR 0.42, 95% CI 0.29–0.62, *p* < 0.001) (Figure 2, Table 3). These findings suggest that earlier initiation of CAPTEM may confer a meaningful survival advantage, potentially reflecting better patient condition at treatment start, improved disease responsiveness, or both. Collectively, the results support consideration of CAPTEM earlier in the therapeutic sequence.

### 3.3. Impact of Treatment Duration

Treatment duration was also strongly associated with clinical outcomes across the study cohort, demonstrating a clear relationship between longer exposure to CAPTEM and improved survival. Patients who received six or more cycles of CAPTEM had significantly improved survival outcomes compared with those who received fewer than six cycles, suggesting a potential cumulative therapeutic benefit.

Median progression-free survival (PFS) was 18.0 months in the ≥6-cycle group versus 2.0 months in the <6-cycle group (HR 0.22, 95% CI 0.16–0.32, *p* < 0.001), indicating a markedly reduced risk of disease progression with extended treatment duration. This substantial difference highlights the importance of maintaining therapy when clinically feasible and tolerated.

**Figure 3:**
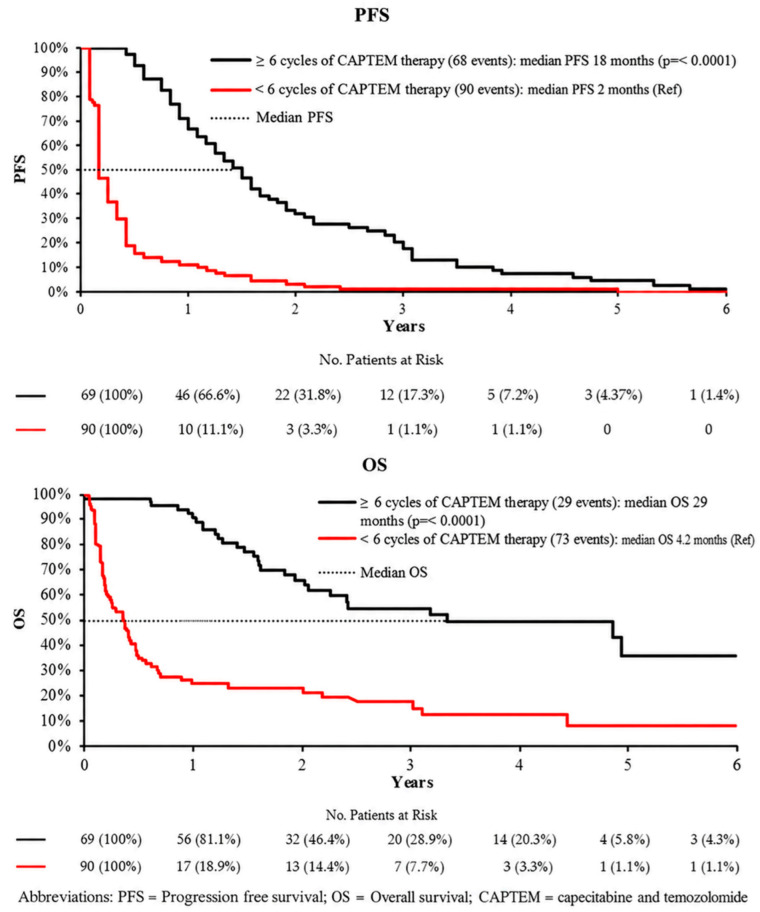
Survival analyses comparing patients receiving greater than 6 cycles of CAPTEM therapy to those with less than 6 cycles of CAPTEM therapy.

Similarly, median overall survival (OS) was significantly prolonged in patients receiving ≥6 cycles, reaching 29.0 months compared with 4.2 months in those receiving fewer cycles (HR 0.22, 95% CI 0.14–0.34, *p* < 0.001) (Figure 3, Table 3). These findings further reinforce the association between longer treatment duration and improved long-term outcomes.

## 4. Discussion

This study represents the largest North American analysis to date evaluating long-term real-world outcomes associated with CAPTEM use in patients with ENENs. By leveraging a broad and heterogeneous cohort reflective of routine clinical practice, our findings provide important insight into treatment effectiveness outside the constraints of controlled clinical trials. Overall, our results demonstrate that CAPTEM yields comparable progression-free survival (PFS) and overall survival (OS) in PNENs and GINENs, while outcomes are significantly inferior in PuNENs and OENENs. Importantly, both first-line use of CAPTEM and receipt of at least six treatment cycles were strongly associated with improved survival outcomes. These observations reinforce the importance of treatment timing and duration in optimizing patient benefit. Collectively, these data support the use of CAPTEM as an effective first-line therapy in appropriately selected patients, extending beyond PNENs to include GINENs, and suggest that its role may be broader than traditionally considered.

The survival outcomes observed in this cohort are notably lower than those reported in the randomized ECOG-ACRIN E2211 trial, which demonstrated a median PFS of 22.7 months and OS of 58.7 months in patients with PNENs [10]. In contrast, median PFS and OS in our study were 9.0 and 19.0 months, respectively. This discrepancy is expected and likely reflects fundamental differences in patient selection, disease characteristics, and treatment context. The E2211 trial enrolled a highly selected population with excellent performance status (ECOG 0–1) and predominantly low- to intermediate-grade disease, conditions that are well known to be associated with more favorable outcomes. In contrast, our analysis captures a broader, unselected real-world population that likely includes patients with more advanced disease, poorer functional status, and greater comorbidity burden. These factors collectively contribute to inferior observed survival metrics in routine practice. Notably, our results align with previously reported retrospective cohorts, which demonstrate wide variability in outcomes, with median PFS ranging from 5.8 to 19.8 months and OS from 19.2 to 105.8 months in CAPTEM-treated PNENs [15,18,19,20,21]. This variability underscores the heterogeneity of patient populations and highlights the challenges of cross-study comparisons.

Our observation of comparable outcomes between PNENs and GINENs is consistent with prior retrospective studies, which similarly report no significant differences in PFS or OS between these subgroups [15,22]. This finding suggests that tumor site alone, particularly between pancreatic and gastrointestinal origins, may not be the primary determinant of CAPTEM responsiveness. Reported survival outcomes in GINENs vary considerably across studies, with median PFS ranging from 1.8 to 12.7 months and OS from 36.9 to 56.1 months; in our cohort, median PFS and OS were 10.5 and 15.9 months, respectively [15,19,22,23]. These differences may reflect variability in tumor biology, grade distribution, prior therapies, and sample size across studies.

In contrast, outcomes in PuNENs remain inconsistent throughout the literature. While some studies have reported no difference relative to PNENs, others—consistent with our findings—demonstrate significantly inferior outcomes [15,22,23]. Reported median PFS and OS in PuNENs range from 13.0 to 33.9 months and 24.4 to 73.9 months, respectively, whereas we observed markedly shorter median PFS and OS of 3.0 and 6.8 months, respectively [15,22,24,25]. These discrepancies may be attributable to differences in patient selection, disease burden, or underlying tumor biology, and they highlight the need for further investigation into this subgroup.

Data for OENENs are limited; prior studies, including those involving unknown primaries, have suggested outcomes comparable to PNENs, in contrast to the substantially poorer outcomes observed in our cohort [15,22]. This divergence may reflect differences in classification, diagnostic approaches, or underlying heterogeneity within the OENEN category. Given the biological heterogeneity of ENENs and the relative paucity of North American data, this degree of variability across studies is not unexpected. Importantly, our findings emphasize the need for more granular, prospective research to better define optimal treatment strategies across ENEN subtypes and to identify predictive factors that may guide individualized therapy selection.

An important consideration when interpreting outcomes across ENEN subtypes is tumor proliferative index, most commonly assessed by Ki-67, which has consistently been shown to correlate with tumor biology, treatment responsiveness, and overall prognosis. Notably, the majority of patients in our analysis had a Ki-67 > 3%, indicating a cohort enriched for intermediate- to high-grade disease and distinguishing it from many previously published studies. Indeed, prior studies evaluating CAPTEM have largely focused on low-grade neuroendocrine tumors, which may partially explain the more favorable outcomes reported in those settings. For example, some series, including the Turkish cohort [15], restricted inclusion to low-grade NETs, while others, such as the study by Spada et al. [22], included a majority of low- to intermediate-grade tumors. In contrast, our real-world cohort reflects a population with higher proliferative indices, including a substantial proportion of grade 2, grade 3 and higher tumors, which may contribute to the comparatively shorter PFS and OS observed.

More recent data further support a role for CAPTEM in higher-grade tumors. A 2025 study by Melhorn et al. demonstrated meaningful clinical benefit in patients with G3 NETs, with survival outcomes that more closely approximate those seen in our analysis [26]. Additionally, another recent study reported that CAPTEM may be more effective than PRRT in high-grade NETs, suggesting a potential therapeutic advantage in this subgroup [27]. Conversely, in neuroendocrine carcinomas (NECs), existing evidence has generally shown limited efficacy of CAPTEM [11,28]. However, given the heterogeneity of high-grade neoplasms and the signals observed in our dataset, further prospective investigation is warranted to better define the role of CAPTEM across the Ki-67 spectrum.

The association between first-line CAPTEM use and improved survival observed in this study is consistent with existing evidence and reinforces the importance of early therapeutic intervention in ENENs. Prior analyses have demonstrated superior response rates and improved survival outcomes when CAPTEM is administered earlier in the disease course, rather than being reserved for later lines of therapy [15,19]. This may reflect a combination of factors, including lower tumor burden, better patient performance status, and increased treatment tolerability at earlier stages of disease. Our findings, therefore, add to the growing body of literature supporting CAPTEM as an effective early-line option and highlight the clinical relevance of thoughtful treatment sequencing. In particular, these data support consideration of CAPTEM as a first-line option in selected ENEN populations, including those beyond PNENs, where appropriate clinical judgment is applied.

Similarly, treatment duration appears to be a critical determinant of outcome in patients receiving CAPTEM. Patients who received at least six cycles of therapy experienced substantially improved progression-free survival (PFS) and overall survival (OS) compared with those receiving fewer cycles, suggesting that sustained exposure to therapy may be necessary to achieve maximal therapeutic benefit. This observation is concordant with prior work, including a study by Melhorn et al., and further emphasizes the importance of maintaining treatment when feasible and clinically appropriate [29]. It is possible that longer treatment duration reflects both favorable disease biology and adequate tolerability, both of which may contribute to improved outcomes.

The treatment landscape for advanced ENENs continues to evolve, particularly with the emergence of peptide receptor radionuclide therapy (PRRT) using 177Lu-DOTATATE. The NETTER-1 trial established PRRT as an effective therapy in midgut ENENs, demonstrating significant improvements in both PFS and OS compared with high-dose octreotide [8]. These advances have expanded the range of therapeutic options available and underscore the importance of individualized treatment selection.

An additional factor that warrants consideration in interpreting our findings is the line and type of therapies administered prior to CAPTEM initiation. In real-world practice, many patients with ENENs receive multiple prior lines of systemic therapy, including somatostatin analogs (SSA), targeted agents such as everolimus and sunitinib, and cytotoxic regimens including oxaliplatin-based chemotherapy. The sequencing of these therapies may influence both tumor biology and subsequent responsiveness to CAPTEM. Recent data from the CABINET trial further inform this landscape, particularly in pancreatic and lung/thymic neuroendocrine tumors, demonstrating the activity of cabozantinib in previously treated patients and highlighting the expanding range of effective later-line options [30]. In addition, the MAVERIC trial showed that everolimus may provide clinical benefit as a maintenance strategy in high-grade GINETs and large cell neuroendocrine carcinoma (LCNEC), suggesting a potential role for treatment continuation or sequencing strategies even in more aggressive disease subsets [31]. Together, these findings underscore the complexity of treatment pathways and the need to contextualize CAPTEM outcomes within prior therapy exposure.

Recent prospective data further inform the evolving role of peptide receptor radionuclide therapy (PRRT) in ENENs. The NETTER-2 trial evaluated first-line 177Lu-DOTATATE in patients with advanced, grade 2–3, somatostatin receptor (SSTR)-positive gastroenteropancreatic NETs and demonstrated a significant improvement in progression-free survival compared with high-dose somatostatin analog therapy [32]. These findings represent a major shift in the treatment paradigm, supporting earlier integration of PRRT in appropriately selected patients and, in particular, suggesting that first-line PRRT may be favored in SSTR-positive tumors with more aggressive features. As a result, there is increasing momentum to move PRRT into earlier lines of therapy rather than reserving it for later use. Ongoing studies such as the COMPOSE trial are expected to further clarify the optimal sequencing of PRRT relative to other systemic therapies, including chemotherapy and targeted agents, and may help define which patient subgroups derive the greatest benefit from early PRRT integration [33].

More recently, combination strategies incorporating CAPTEM and PRRT have shown promising activity and are increasingly being explored as a means of improving therapeutic outcomes in ENENs. For example, di Santo et al. reported higher response rates with combined CAPTEM and 177Lu-DOTATATE compared with either modality alone, suggesting a potential synergistic interaction between systemic chemotherapy and targeted radionuclide therapy, although the study was underpowered to detect survival differences [34]. These results are particularly relevant in the context of more aggressive disease, where single-modality treatment may be insufficient to achieve durable control. Furthermore, such approaches may help overcome resistance mechanisms or enhance tumor sensitivity to PRRT. These findings highlight the need for further investigation into optimal sequencing and combination strategies integrating CAPTEM and PRRT in real-world settings.

### Study Limitations

Several limitations should be acknowledged when interpreting the results of this study. The retrospective design inherently introduces the potential for selection bias and residual confounding, as treatment decisions and patient characteristics were not controlled in a prospective manner. Although this represents the largest North American cohort of CAPTEM-treated ENENs to date, the overall sample size remains insufficient to support detailed subgroup analyses stratified simultaneously by tumor site, treatment line, and treatment duration. As such, some clinically relevant differences may not have been fully captured. Future studies with larger datasets and prospective designs will be required to more robustly address these important questions and validate our findings.

Additionally, the inclusion of a heterogeneous patient population, while reflective of real-world clinical practice, may contribute to variability in observed outcomes. Patients in this cohort likely differed in disease biology, tumor grade, comorbidities, and prior treatment exposures, all of which may influence both treatment response and survival.

Furthermore, toxicities were not systematically captured in this analysis due to inconsistent documentation across clinical notes, lack of contemporaneous laboratory data at the time of reported adverse events, and the absence of standardized definitions of toxicity in the retrospective setting. This limits the ability to fully evaluate the safety profile of CAPTEM in this population.

In this study, CAPTEM was primarily utilized as a later-line therapy. Patients had frequently received prior treatments, including somatostatin analogues, peptide receptor radionuclide therapy (PRRT), or targeted molecular agents earlier in their disease course. These prior therapies may have confounded progression-free survival (PFS) outcomes, particularly in subgroup analyses stratified by timing of CAPTEM initiation (earlier versus later lines of therapy).

The heterogeneity of the study population constitutes an additional limitation, as the relatively small number of patients within each subgroup may limit statistical power and affect the reliability and interpretability of subgroup-specific outcomes.

Nevertheless, the use of comprehensive, population-based data derived from high-quality provincial databases represents a key strength of this study and enhances the generalizability of these findings to broader real-world populations.

## 5. Conclusions

In this real-world cohort, CAPTEM demonstrated clinically meaningful activity beyond PNENs, particularly in GINENs, supporting its broader applicability across diverse ENEN subtypes encountered in routine practice. These findings reinforce the potential versatility of CAPTEM as a systemic therapy option and suggest that its benefit is not limited to pancreatic primaries alone. Treatment duration of at least six cycles appeared to be strongly associated with improved outcomes, underscoring the importance of sustained therapy exposure when clinically feasible and well tolerated. In addition, our results indicate that CAPTEM may be reasonably considered in earlier lines of therapy in carefully selected patients, where improved performance status and lower disease burden may enhance treatment benefit.

However, outcomes varied significantly across tumor subtypes, highlighting the substantial biological heterogeneity that characterizes ENENs and the need for more refined, individualized treatment strategies. In particular, poorer outcomes observed in PuNENs and OENENs emphasize the importance of improved patient selection and the development of subtype-specific therapeutic approaches. Given the retrospective nature of this analysis and the potential for confounding factors, prospective studies are warranted to better define optimal patient selection, treatment sequencing, and duration of CAPTEM therapy.

Taken together, these findings provide compelling real-world evidence supporting the integration of CAPTEM earlier in the treatment paradigm and across a broader spectrum of ENENs, while also underscoring the urgent need for prospective, biomarker-driven studies to optimize outcomes in this heterogeneous and challenging disease population.

## Figures and Tables

**Table 1:** Baseline patient, tumor, and treatment characteristics of whole population.

Characteristic			Patients with ENENs (*n* = 159)
**Age (years)**			
	Median		58.0
	Mean (SD; range)		57.4 (13.1; 20–83)
**Sex [*n* (%)]**			
	Male		95 (59.8%)
	Female		64 (40.2%)
**Tissue of Origin [*n* (%)]**			
	Pancreas (PNEN)		67 (42.1%)
	Other GI (GINEN)		38 (24.5%)
		Gastroesophageal	5 (3.1%)
		Duodenal/Jejunal	8 (5.0%)
		Ileal/Ileocecal	18 (11.3%)
		Colorectal	7 (4.4%)
	Pulmonary (PuNEN)		35 (20.1%)
	Other (OENEN)		19 (13.2%)
		Unknown Primary OENEN	12 (7.5%)
		Thyroid OENEN	3 (1.9%)
		Genitourinary OENEN	2 (1.3%)
		Skin/Mucosal OENEN	2 (1.3%)
**Histology [*n* (%)]**			
	Large cell neuroendocrine		12 (7.6%)
	Small cell		19 (12.0%)
	Carcinoid		49 (30.8%)
	Neuroendocrine		36 (22.6%)
	Atypical carcinoid		32 (30.1%)
	Other *		11 (6.9%)
**Ki-67 Status [*n* (%)]**			
	<3%		45 (28.3%)
	3% to 20%		54 (34.0%)
	>20%		60 (37.7%)
**Overall Staging AJCC-6/7/8 Clinical Criteria [*n* (%)]**			
	I		4 (2.5%)
	II		9 (6.7%)
	III		15 (9.4%)
	IV		131 (82.4%)
**CAPTEM Line Used [*n* (%)]**			
	First		101 (63.5%)
	Second		48 (30.2%)
	Third		8 (5.0%)
	Fourth		1 (0.6%)
	Fifth		1 (0.6%)
**Number of CAPTEM Cycles (Number)**			
	Median		5.0
	Mean (SD, range)		8.1 (9.9; 1–69)
	<6 CAPTEM cycles (%)		90 (56.6%)
	≥6 CAPTEM cycles (%)		69 (43.4%)

* Other types include Islet cell, Somatostatinoma, Insulinoma, Composite carcinoid, Merkel cell, and Medullary thyroid carcinoma. Abbreviation: CAPTEM = capecitabine and temozolomide.

**Table 2:** Baseline patient, tumor, and treatment characteristics by tissue of origin.

Characteristic		PNENs (*n* = 67)	GINEN (*n* = 38)	PuNEN (*n* = 35)	OENEN (*n* = 19)	*p*-Value *
**Age (years)**						
	Median	55.0	61.0	60.0	60.0	0.598
	Mean (SD; range)	56.3 (13.7; 25–82)	58.7 (12.0; 20–77)	59.0 (11.8; 34–83)	55.4 (15.3; 27–78)	
**Sex [*n* (%)]**						
	Male	42 (62.7%)	18 (47.4%)	24 (68.6%)	11 (57.9%)	0.281
	Female	25 (37.3%)	20 (52.6%)	11 (31.4%)	8 (42.1%)	
**Histology [*n* (%)]**						
	Large cell neuroendocrine	2 (3.0%)	2 (5.3%)	7 (20.0%)	1 (5.3%)	<0.001
	Small cell	0 (0%)	1 (2.6%)	16 (45.7%)	2 (10.5%)	
	Carcinoid	23 (34.3%)	21 (55.3%)	1 (2.9%)	4 (21.0%)	
	Neuroendocrine	19 (28.4%)	7 (18.4%)	4 (11.4%)	6 (31.7%)	
	Atypical carcinoid	17 (25.4%)	6 (15.8%)	7 (20.0%)	2 (10.5%)	
	Other	6 (8.9%)	1 (2.6%)	0 (0%)	4 (21.0%)	
**Ki-67 Status [*n* (%)]**						
	<3%	19 (28.4%)	15 (39.5%)	6 (17.1%)	5 (26.3%)	0.060
	3% to 20%	29 (43.2%)	11 (28.9%)	10 (28.6%)	4 (21.1%)	
	>20%	19 (28.4%)	12 (31.6%)	19 (54.3%)	10 (52.6%)	
**Overall Staging AJCC-6/7/8 Clinical Criteria [*n* (%)]**						
	I	1 (1.5%)	0 (0%)	2 (5.7%)	1 (5.3%)	0.002
	II	8 (11.9%)	0 (0%)	1 (2.9%)	0 (0%)	
	III	4 (6.0%)	1 (2.6%)	9 (25.7%)	1 (5.3%)	
	IV	54 (80.6%)	37 (97.4%)	23 (65.7%)	17 (89.4%)	
**CAPTEM Line Used [*n* (%)]**						
	First	45 (67.2%)	27 (71.0%)	20 (54.2%)	9 (47.4%)	0.406
	Second	18 (26.9%)	9 (23.7%)	12 (34.2%)	9 (47.4%)	
	Third	4 (6.0%)	2 (5.3%)	1 (2.9%)	1 (5.2%)	
	Fourth	0 (0%)	0 (0%)	1 (2.9%)	0 (0%)	
	Fifth	0 (0%)	0 (0%)	1 (2.9%)	0 (0%)	
**Number of CAPTEM Cycles (Number)**						
	Median	6.0	5.0	3.0	3.0	0.109
	Mean (SD, range)	9.0 (10.4; 1–56)	10.1 (12.6; 1–69)	5.5 (5.9; 1–25)	5.5 (6.5; 1–22)	
	<6 CAPTEM cycles (%)	31 (46.3%)	20 (52.6%)	25 (71.4%)	14 (73.7%)	0.036
	≥6 CAPTEM cycles (%)	36 (53.7%)	18 (47.4%)	10 (28.6%)	5 (26.3%)	

* To compare continuous variables, Kruskal–Wallis tests were used, and to compare categorical variables, Chi-Squared analysis was used. Abbreviations: CAPTEM = capecitabine and temozolomide; PNENs = Pancreatic neuroendocrine neoplasms; GINENs = Gastrointestinal neuroendocrine neoplasms; PuNENs = Pulmonary neuroendocrine neoplasms; OENENs = Other endocrine and neuroendocrine neoplasms. Other types include Islet cell, Somatostatinoma, Insulinoma, Composite carcinoid, Merkel cell, and Medullary thyroid carcinoma.

**Table 3:** Cox regression analysis for PFS and OS.

Proportional-Hazard Models		PFS			OS		
		HR	95% CI	*p*-Value	HR	95% CI	*p*-Value
Whole population (*n* = 159)	<6 cycles of CAPTEM vs. ≥6 cycles	0.22	0.16–0.32	**<0.001**	0.22	0.14–0.34	**<0.001**
	CAPTEM first line vs. later line	0.56	0.40–0.78	**<0.001**	0.42	0.29–0.62	**<0.001**
PNENs (*n* = 67; ref) vs. GINENs (*n* = 38)	Unadjusted	1.14	0.76–1.71	0.114	1.13	0.69–1.85	0.150
	Adjusted for age and sex	1.10	0.79–1.65	0.101	1.11	0.69–1.81	0.135
PNENs (*n* = 67; ref) vs. PuNENs (*n* = 35)	Unadjusted	1.64	1.10–2.63	**0.023**	1.72	1.06–2.71	**0.015**
	Adjusted for age and sex	1.65	1.15–2.51	**0.021**	1.73	1.08–2.71	**0.014**
PNENs (*n* = 67; ref) vs. OENENs (*n* = 19)	Unadjusted	1.56	1.12–2.58	**0.018**	1.49	1.10–2.73	**0.023**
	Adjusted for age and sex	1.59	1.14–2.74	**0.020**	1.51	1.05–2.80	**0.026**

Abbreviations: PFS = Progression-free survival; OS = Overall survival; CAPTEM = capecitabine and temozolomide; PNENs = Pancreatic neuroendocrine neoplasms; GINENs = Gastrointestinal neuroendocrine neoplasms; PuNENs = Pulmonary neuroendocrine neoplasms; OENENs = Other endocrine and neuroendocrine neoplasms. The bolded *p* values represent statistical significance for the study.

## Data Availability

The raw de-identified data supporting the conclusions of this article will be made available by the authors on request.

## Abbreviations

The following abbreviations are used in this manuscript:

CAPTEM	Capecitabine and Temozolomide
ENEN	Endocrine and Neuroendocrine Neoplasm
PNEN	Pancreatic Neuroendocrine Neoplasm
GINEN	Gastrointestinal Neuroendocrine Neoplasm
PuNEN	Pulmonary Neuroendocrine Neoplasm
OENEN	Other Endocrine and Neuroendocrine Neoplasms
NET	Neuroendocrine Tumor
OS	Overall Survival
PFS	Progression-free Survival
AHS	Alberta Health Services
ACR	Alberta Cancer Registry
ARIA-MO	Cancer Centre Electronic Medical Record
DAD	Discharge Abstract Database
NACRS	National Ambulatory Care Reporting System
DPYD	Dihydropyrimidine Dehydrogenase
MGMT	O6-Methylguanine-DNA Methyltransferase
